# Low-Cost Wearable Band Sensors of Surface Electromyography for Detecting Hand Movements

**DOI:** 10.3390/s22165931

**Published:** 2022-08-09

**Authors:** Manuela Gomez-Correa, David Cruz-Ortiz

**Affiliations:** 1 Medical Robotics and Biosignal Processing Laboratory, Unidad Profesional Interdisciplinaria de Biotecnología, Instituto Politécnico Nacional, Mexico City 07340, Mexico; 2 Facultad de Ingeniería, Universidad de Antioquia, Medellin 050010, Colombia

**Keywords:** surface electromyography, wearable armband, multichannel system, wireless communication, artificial neural networks

## Abstract

Surface electromyography (sEMG) is a non-invasive measure of electrical activity generated due to muscle contraction. In recent years, sEMG signals have been increasingly used in diverse applications such as rehabilitation, pattern recognition, and control of orthotic and prosthetic systems. This study presents the development of a versatile multi-channel sEMG low-cost wearable band system to acquire 4 signals. In this case, the signals acquired with the proposed device have been used to detect hand movements. However, the WyoFlex band could be used in some sections of the arm or the leg if the section’s diameter matches the diameter of the WyoFlex band. The designed WyoFlex band was fabricated using three-dimensional (3D) printing techniques employing thermoplastic polyurethane and polylactic acid as manufacturing materials. Then, the proposed wearable electromyographic system (WES) consists of 2 WyoFlex bands, which simultaneously allow the wireless acquisition of 4 sEMG channels of each forearm. The collected sEMG can be visualized and stored for future post-processing stages using a graphical user interface designed in Node-RED. Several experimental tests were conducted to verify the performance of the WES. A dataset with sEMG collected from 15 healthy humans has been obtained as part of the presented results. In addition, a classification algorithm based on artificial neural networks has been implemented to validate the usability of the collected sEMG signals.

## 1. Introduction

The development of wearable devices has seen remarkable growth, considering that worldwide the use of wearable devices will have a compound annual growth rate of 38% in the period from 2017 to 2025 [1]. Indeed, for the year 2019, it was estimated that about 225 million portable devices were sold [2]. In general, the design of wearable systems is based on the incorporation of intelligent sensors, artificial intelligence, the internet of things (IoT), and big data, in such a way that it is possible to obtain information of interest from the human body [3,4,5]. In that sense, physiological signals such as blood pressure, oxygen saturation, electrocardiography, and electromyography (EMG) signals are some of the essential information measured by using devices such as watches, pendants, bracelets, and other types of accessories [5].

Particularly, EMG signals describe the electrical activity generated during the muscle contraction processes. These signals have been used in research and clinical areas for various applications such as diagnosing neuromuscular diseases [6], evaluating muscle or nerve damage [7], and the biomechanical analysis of movements [8,9]. Currently, EMG signals can be measured by invasive methods, which use needle electrodes, and non-invasive techniques that consider surface electrodes placed on the person’s skin [8].

Mainly, surface electromyography (sEMG) signals have been extensively used in developing prosthetic devices [10], technology based on human–machine interaction [11], and equipment used for rehabilitation [12]. Therefore, elements such as bracelets or armbands have been developed to obtain sEMG signals that provide relevant information in a non-invasive way. These armbands generally have several EMG sensors located radially on a flexible band, which is easy to place and can be used in several applications [13], such as recognition of hand gestures [14], the control of prostheses [15], video games [16], and therapy monitoring [17].

One of the most important developments of this type of device was the Myo band created by Thalmic Labs, which consists of 8 electrodes, a 9-axis inertial measurement unit, and a Bluetooth Low Energy module. Moreover, the Myo armband weighs 93 g and has a thickness of 1.15 cm. Despite the various applications in which it was implemented, this armband has disadvantages, such as a sampling frequency of 200 Hz, which leads to the loss of relevant information since the dominant range of the sEMG signals is from 10 to 500 Hz [12]. Due to this, the Myo band is not the best option in applications such as classification or detecting movements. Furthermore, it is currently discontinued [13]. Here, it should be highlighted that even though the Myo band is currently discontinued, its resale price is around USD 475.

In 2018, Mahmoud Tavakoli et al. [18] developed a minimalist band with a sampling frequency of 1000 Hz, which used only two sensors for the recognition of four gestures (open, close, wave in, wave out). Nevertheless, the application of said bracelet is reduced due to the number of movements considered. Additionally, the optimal positioning of the sensors varies for each subject, generating relevant deviations in the obtained results. Moreover, even though the system only had two channels, the design was not sufficiently compact and did not operate wirelessly, which makes it challenging to implement in real scenarios.

Another sEMG armband developed was the 3DC Armband proposed by the Biomedical Microsystems Laboratory at Laval University [19], which has 10 channels and a 9-axis inertial unit. In addition, it has a sampling frequency of 1000 Hz with a 10-bit ADC and a weight of 62 g. In addition, this device has a height of 3.7 cm, a thickness of 1.6 cm, and a cost of approximately USD 150, considering that the price of the System-on-Chip was changed by the cost of a comparable product. Despite having a low cost, this device has a highly complex system and a non-standardized manufacturing process due to the proposed joining elements for the sensor boxes and the complexity of the sensors used, which, although they offer good characteristics, restrict its reproducibility. Likewise, OYMotion Technologies developed the gForce EMG Armband, which has 8 channels for sEMG signals acquisition, a 9-axis inertial unit, and Bluetooth data transmission. Additionally, the gforce band weights 80 g, it has a maximum frequency of 1000 Hz for an 8-bit ADC, and its dimensions correspond to a perimeter of 20–28 cm, a height of 4 cm, and a thickness of 1 cm. However, this system requires a USB module for data transmission to a computer, which increases the system’s complexity, and its high cost (USD 1250), compared to the solutions above, reduces its affordability [19].

The present paper describes the design of a wearable electromyographic system (WES), which consists of two armbands, called WyoFlex, to acquire four sEMG signals. The WyoFlex band consists of four sEMG sensors measured by using a 12-bit ADC module of a FireBeetle ESP32-E microcontroller. The WyoFlex band considers a data transmission system through Wi-Fi to send and visualize the rebuilt signals online. Then, a maximum sampling frequency of 1600 Hz is guaranteed to avoid the loss of relevant information in the acquired sEMG signals. Furthermore, as one of the main characteristics, the proposed device has a mechanical design completely developed in three-dimensional (3D) printing, thus achieving a functional and affordable device. Finally, to evaluate its performance, the device was implemented to recognize six hand gestures by applying an algorithm based on artificial neural networks (ANNs). Notice that in this case, the signals acquired with the proposed device have been used to detect hand movements. However, the WyoFlex band could be used in some sections of the arm or the leg if the section’s diameter matches the diameter of the WyoFlex band.

The main novelties of the proposed design are:

- A novel WES composed of two wearable armbands called WyoFlex. The structural design of each armband is characterized by its flexibility and easy manufacturing process, which is based on additive manufacturing techniques such as 3D printing.
- The four sEMG signals are acquired by using dry electrodes, which guarantee the portability of the proposed device. Additionally, the proposed WyoFlex band is self-contained, since all the elements integrating its electronic instrumentation aside from its power supply are attached to the band.
- A graphical user interface (GUI) for the WyoFlex armband allows the acquisition of eight sEMG signals (four per armband) with a maximum sampling frequency of 1600 Hz. The proposed GUI considers the User Datagram Protocol (UDP) as part of the implemented wireless communication.
- A database integrated by sEMG signals of 15 healthy volunteers who performed six hand movements has been obtained. The database was used to test a hand gesture recognition algorithm based on the implementation of an ANN, which served to validate the functionality of each WyoFlex device.

## 2. System Design

The proposed WES is integrated by two WyoFlex bands. Each armband was designed to obtain sEMG signals from the forearm when the user executes different hand movements such as flexion, extension, ulnar deviation, radial deviation, power grip, and lateral grip [20]. The main characteristics expected in the proposed WyoFlex band are that the device should be dimensionally portable (wearable), ergonomic, and easy to implement.

The structural characteristics also must include the total incorporation of the elements integrating the device and their efficient fastening. The previous idea leads to considering different instrumentation criteria, such as selecting electronic components (that should not be robust) with technical characteristics that allow the design of a wireless and safe WES. The design concept of each WyoFlex band is explained in detail below in the subsequent paragraphs.

### 2.1. Structural Design

**Figure 1:**
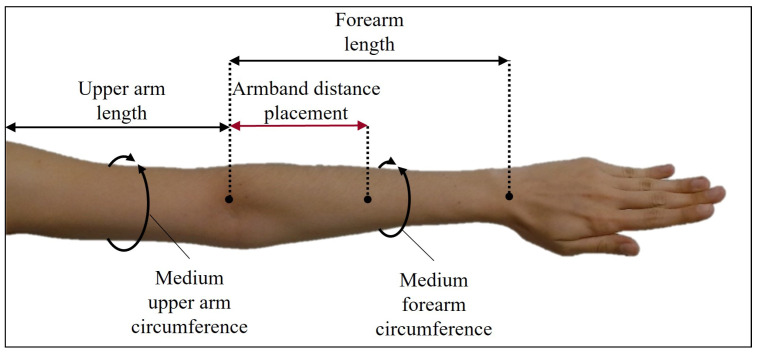
Anthropometric dimensions considered in the WyoFlex design.

The first criteria for designing each WyoFlex band was the selection of the structural dimensions that it should have. Then, the forearm anthropometric measurements average obtained from the Latin American population participating in this project was selected to define the structural dimensions (a detailed description of the subjects is provided below in Section 3.2). Figure 1 shows the dimensions considered, where the medium upper arm circumference average is 29 cm. In contrast, the medium forearm circumference average is 23.5 cm.

Notice that the proposed design considers only WyoFlex bands for the forearm. Then, the medium forearm circumference average was the central dimension. However, the medium upper arm circumference average is mentioned in the scheme since this measure could be helpful in future stages of this research. Aside from the average anthropometric measurements, the proposed structural design also considers the dimensions of the electronic elements integrating the armband. In this case, each of them includes four sEMG sensors, a microcontroller, a battery, structural elements to support the components, and parts for fastening the band to the forearm. Keeping all this in mind, a computer-aided design (CAD) model made in the software CAD Inventor ^®^ 2021 from Autodesk (San Rafael, California, USA) is proposed.

**Figure 2:**
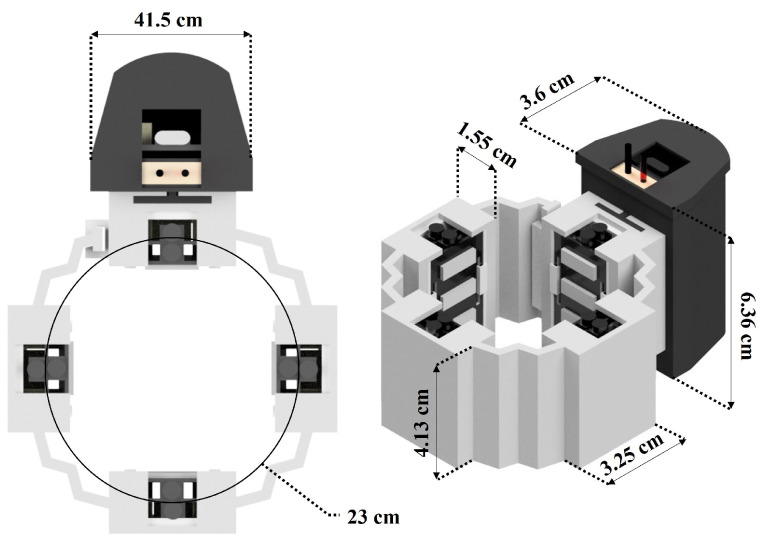
General dimensions of the armband.

Figure 2 shows the general dimensions considered for the WyoFlex band fabrication. Here, the main criteria is the central diameter considered for the armband, which was selected as 23 cm. Notice that this dimension satisfies the medium forearm circumference average (23.5 cm) aside from ensuring that the sensors in the bracelet are in touch with the participant’s skin.

**Figure 3:**
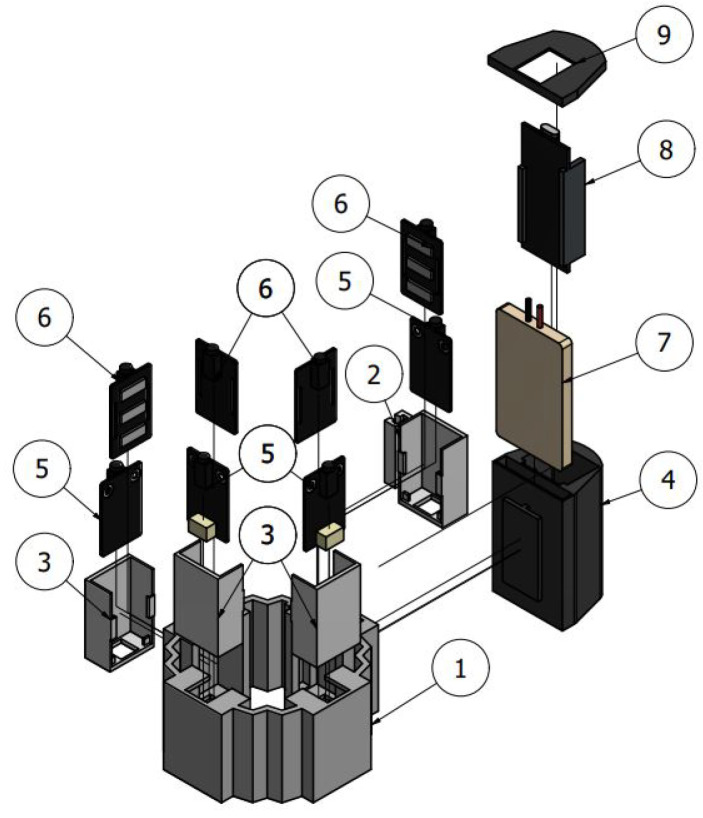
Structural design and elements integrating the WyoFlex band. (1) Flexible band, (2 and 3) Sensor case, (4) Control system case, (5 and 6) EMG sensor, (7) Battery, (8) FireBeetle-E microcontroller, (9) Control system cover.

Figure 3 shows an exploded view of the WyoFlex band CAD model, aiming to enhance the spatial location of each component. Then, a detailed description of each element integrating the WyoFlex is presented below, with the bold numbers at the beginning of the paragraphs denoting the components in the mentioned figure.

**Flexible band (1):** This component is the main structural element of the device. It consists of a flexible band manufactured by 3D printing with Thermoplastic Polyurethane (TPU), which is a flexible material that, in addition to allowing the incorporation of the rest of the elements, facilitates the adaptation of the device to the user’s forearm. The flexible band has four compartments for the sEMG sensors and a support mechanism for holding the case that contains the battery and the microcontroller.

**Sensor case (2 and 3):** This case is made of Polylactic Acid (PLA). Its main function is the correct and stable fastening of the modules that make up the EMG sensors and guarantee the contact of the electrodes with the skin. In particular, the case part denoted by the number 2 in Figure 3 has a slider system that allows the bracelet to be easily placed on the person’s forearm.

**Control system case (4):** This element aims to fasten the battery that powers the entire system and the microcontroller to the structure. It is made of PLA, and its resistance ensures the integrity of the components stored in the armband.

**EMG sensor (5 and 6):** These modules correspond to the sensor implemented in the manufacture of the WyoFlex band. A detailed description of the sensors has been presented in the electronic instrumentation subsection.

**Battery (7):** This is used as power supply for all the electronic components of the system.

**FireBeetle ESP32-E microcontroller (8):** The microcontroller is a DFRobot development, which was specifically designed for IoT applications.

**Control system cover (9):** This element made of PLA has the function of keeping the microcontroller and the battery attached to the flexible band.

### 2.2. Electronic Instrumentation

As previously mentioned, the armbands integrating the WES contain several electronic devices, such as sEMG sensors, a microcontroller, and a battery, as part of their electronic instrumentation. Then, a detailed description of the electrical characteristics of each component is provided.

**sEMG sensor:** The Gravity Analog EMG sensor has been launched by the cooperation of DFRobot and OYMotion. It comprises a module containing the electrodes, and a module that integrates a filtering and amplification circuit (items 5 and 6 in Figure 3, respectively). It has an analog output whose output voltage varies between 0 and 3 V, and its reference voltage corresponds to 1.5 V. This sensor allows sEMG signals to be obtained non-invasively and is made up of dry metal electrodes, which gives them a longer lifespan and ease of use.

**Battery:** A rechargeable 3.7 V lithium-polymer (Li-Po) battery with a current of 1200 mAh is implemented in this model.

**FireBeetle ESP32-E microcontroller:** The microcontroller is a DFRobot development, which contains an ESP-WROOM-32E board with dual-core chips and allows communication by both Wi-Fi and Bluetooth. Furthermore, it contains an integrated circuit for charging Li-Po batteries. This microcontroller has low power consumption and is compatible with Arduino IDE. Moreover, due to its small size, it is widely used in wearable applications.

**Figure 4:**
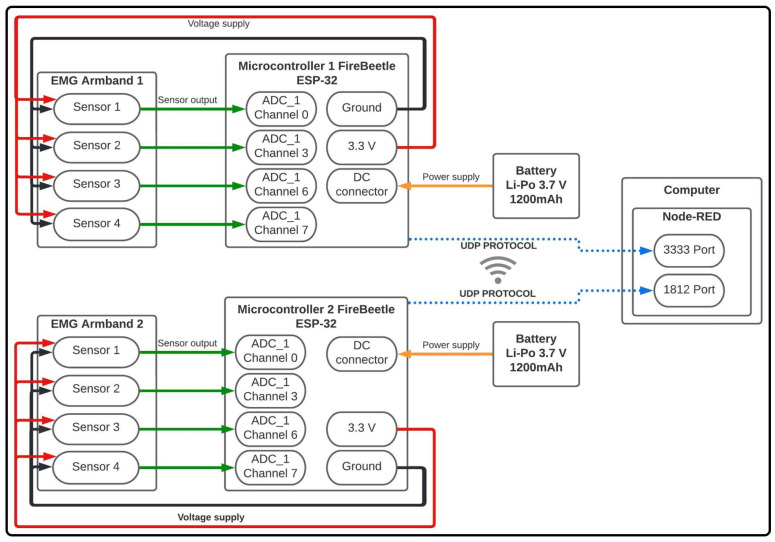
General electronic instrumentation of the WES.

Particularly, the electronic instrumentation of the proposed WES is divided into three main parts: the sensors’ connection with the microcontroller, the electrical power supply system, and Wi-Fi communication. Figure 4 shows a general diagram to evidence the electronic instrumentation as well as the interconnections between each element.

In the first place, a 3.7 V Li-Po battery was used to power the system, which directly fed the microcontroller through a direct current connector (see the solid yellow lines in Figure 4). From the above, a 3.3 V output pin and a ground pin of the ESP-32 (solid red and black lines, respectively) were used to power the 4 sEMG sensors integrating the armband.

Then, a direct connection (solid green lines) between the sEMG sensors and the channels of module 1 of the analog–digital converter (ADC) in the microcontroller was used. On the other hand, for the connection of the microcontroller with the GUI based on the Node-RED environment (described below in Section 2.3) in a external computer, the UDP was implemented, explicitly using ports 3333 and 1812. Finally, through the GUI, data were received for later storage and visualization.

### 2.3. Wireless System and Graphical User Interface Design

**Figure 5:**
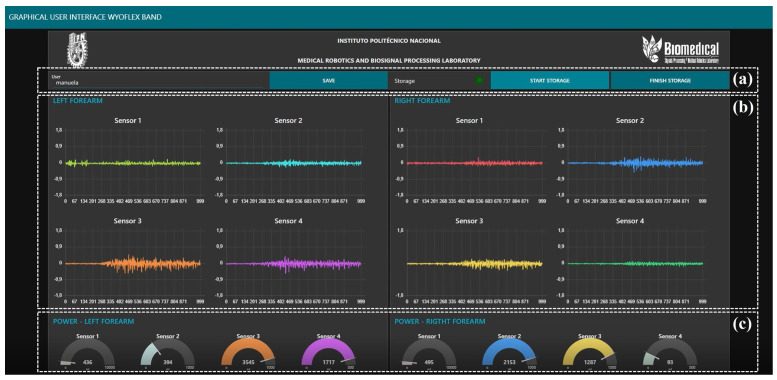
Graphical user interface developed in Node-RED. (**a**) Dialog box, (**b**) Plots for sEMG signals visualization, (**c**) Plots of sEMG signals power.

The UDP was the method used for the process of sending data. Accordingly, because a WyoFlex band was implemented for each forearm, a different UDP port was established for sending data to the microcontroller of each device. The selected ports were 3333 and 1812. Then, a GUI was developed in the Node-RED tool for the reception, visualization, and storage of the information, implementing Wi-Fi communication between the microcontroller and the web application. Figure 5 shows all the elements considered in the designed GUI.

The GUI allows the information acquired from the 2 WyoFlex bands to be displayed and stored. Notice that all the signals visualized in the GUI consider the data on-site, since no one processing is applied over the visualized signals. The proposed GUI can be divided into three main sections. In Figure 5a), the GUI has a dialog box, a LED, and three buttons for the storage process. With the dialog box, the users can give a name to the CVS file where the data will be stored. The provided file name will only be used if the user presses the “Save” button. If not, the test is saved with a name established in the interface settings. To confirm the successful saving of the file, a notification will appear on the right side of the screen indicating whether the name is saved. Then for the storage to start, the user needs to press the “Start Storage” button; the GUI will only store the data sent after the user press this button. To finish the process, it is necessary to press the “Finish Storage” button, and a notification will appear again to indicate the successful saving data process. The generates file contains a vector with the information of the eight sensors separate with semicolons. Additionally, the GUI has an LED that indicates to the user whether storage is enabled (green light) or if the data is not being stored (red light). Figure 5b) shows the plots where the visualization process is executed, which in this case consists of four graphs per WyoFlex band where the last 1000 samples obtained with each sensor are shown (left forearm on the left and right forearm on the right). On the other hand, the last section of the GUI is presented in Figure 5c), where eight plots are shown and each plot shows the power of each sEMG signal in mJ.

### 2.4. Affordability of the WyoFlex Band

Once all the elements integrating the WyoFlex band have been described, the most relevant aspects of the proposed device and some commercial sEMG bracelets presented in the literature have been summarized in Table 1 to corroborate the affordability of the WyoFlex band.

## 3. Method

### 3.1. Fabrication of the Wearable Band Sensor

The manufacturing method used for each armband was 3D printing. Here, the Creality Ender 3 Pro printer was used for both materials; the flexible material parts (TPU) and pieces made of rigid material (PLA). For the manufacture of the PLA cases, which contained the sensors and the microcontroller, used a printing speed of 50 mm/s, a filling density of 20%, and a printing temperature of 200 °C. On the other hand, for the band printed on flexible material the parameters were a printing speed of 15 mm/s, fill density of 20%, and a printing temperature of 235 °C. During the printing process, it was observed that the minimum thickness of the flexible armband walls had to be 2 mm to avoid failure, while the PLA pieces could have a minimum thickness of 0.5 mm. Table 2 shows the printing times of each elements of the armband. Then, it can be concluded that a complete set of pieces of the armband can be obtained in less than 38 h.

### 3.2. Subjects

The data set considered in this study was collected from 15 healthy human subjects (11 males and 4 females, aged from 19 to 37 years, mean age of 26.7 years, all right-handed). All the participants previously signed an informed consent form as part of this research project. Then, all participants agreed to take part in the experiments. The study protocol underwent review and approval by the Secretaria de Investigacion y Posgrado del IPN, which is in charge of approving the research protocols according to the ethical standards defined in the declaration of Helsinki.

### 3.3. WyoFlex Location in the Forearm

As part of the acquisition protocol for measuring the sEMG signals, anatomic references should be provided to indicate the correct location of the sensor [18,21,22,23,24]. Then, to reproduce the electrode positions, European recommendations for sEMG (SENIAM) were followed [25]. Therefore, each WyoFlex band was placed approximately in the middle of the forearm, verifying the electrodes made enough contact with the skin to avoid extra noise.

**Figure 6:**
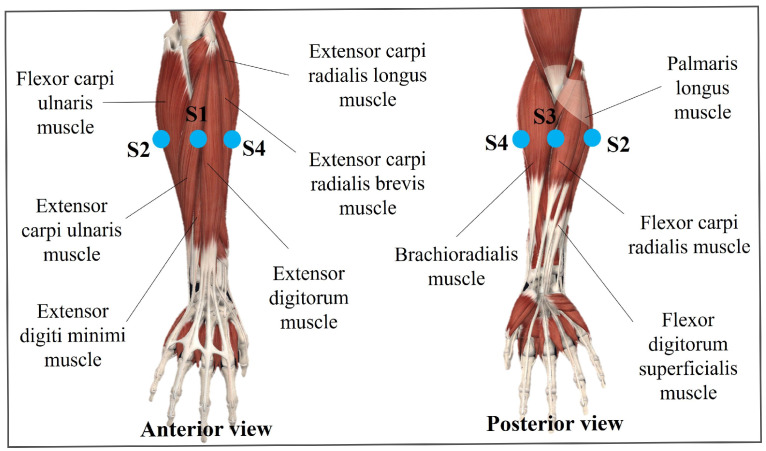
Location of the sEMG sensors in the forearm.

The WyoFlex was located with the cables’ outputs in the upper part of the forearm and, we placed sensor 1 (sensor located under the microcontroller case) in the anterior part of the forearm, specifically on the Extensor digitorum muscle and the Extensor carpi ulnaris muscle. Likewise, channel 2 was located on the external side of the forearm, covering the Palmaris longus muscle and Flexor carpi ulnaris muscle. As a consequence, in the posterior part of the forearm was placed channel number 3, specifically on the Brachioradialis muscle an the Flexor carpi radialis muscle. Finally, the sensor corresponding to channel 4 was over the Extensor carpi radialis longus muscle and the Extensor carpi radialis brevis muscle. Figure 6 shows the location of each sensor, where S1, S2, S3, and S4 denote the sensor 1, sensor 2, sensor 3, and sensor 4, respectively. This figure considers anatomic schemes obtained from the software Complete Anatomy from Elsevier.

### 3.4. sEMG Signals

The sEMG signals were acquired with four Gravity Analog EMG sensors in each WyoFlex band. Each sensor comprises two modules, the first one being called the dry electrode board and the second one the signal transmitter board, which contains the filtering and amplification stages. In this case, the signal transmitter board requires a supply voltage of 3.3–5.5 V, considering an operating voltage of 3 V with a detection range of ±1.5 mV. Then, each sensor provides an analog output voltage, which can vary between 0 and 3 V. The corresponding reference voltage is equal to 1.5 V.

Based on previous works [18,21,22,23,24], each dry electrode board was placed around the circumference of the forearm as previously explained in Section 3.3. Each sEMG sensor was sampled at a rate of 1000 Hz (considering that the maximum sample rate of the WyoFlex is 1600 Hz) with the 12-bit ADC module of the FireBeetle ESP32- microcontroller. Before the study, none of the subjects had been trained on sEMG recording. During the recording of the sEMG signals, each subject sat on a chair in front of a computer with the proposed GUI screen to view all the sEMG signals. At the same time, a video tutorial was displayed to indicate the specific sequence of movements that should be executed. The movements considered in the test protocol are described below.

### 3.5. Test Protocol

**Figure 7:**
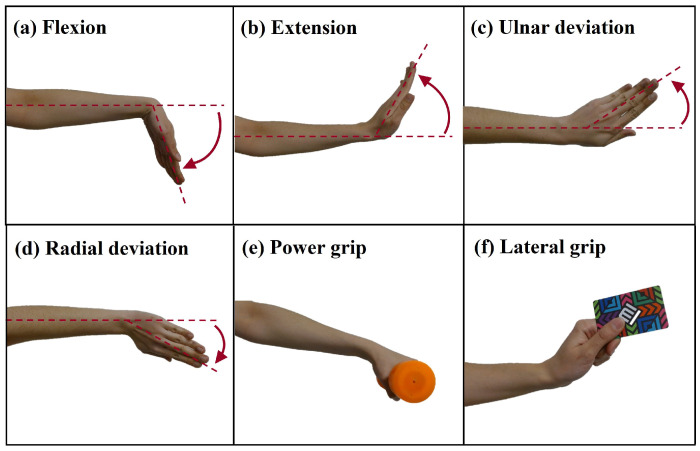
Movements executed in the test protocol.

For data recording with the WyoFlex band, a tutorial was established that continuously showed the actions that the user had to perform during the test. The test consisted of 3 repetitions of the 6 movements of interest (see Figure 7), which allowed the acquisition of 18 signals per test. The execution of each one of the motions had an approximate duration of 10 s. In the first 4 s, the person was shown the movement to perform, the next 3 s were available for the preparation and execution of the movement, and in the last 3 s, the user had to maintain the position of the action [26].

### 3.6. Data Acquisition Protocol

As previously mentioned, in the data acquisition process, a sampling frequency of 1000 Hz was established for each sensor, considering that the dominant range of the sEMG signals is from 10 to 500 Hz [12]. From the above, the data was sent through a message containing a character to identify the start of a new joint message with 4 characters of the ADC value for each sensor, thus obtaining 17-character messages. Equation (1) shows the architecture of the sent vector information denoted by $DS_{j}$.

(1) $$\begin{array}{c} DS_{j}=\left\{A,S1_{j},S2_{j},S3_{j},S4_{j}\right\} \end{array}$$

In (1), $DS_{j}$ denotes the vector information, which contains the data of the 4 acquired signals, whereas the subscript j={I,D} refers to the left and right forearm, respectively. Finally, the data received in Node-RED was separated for each sensor and stored in vectors for the online visualization of the sEMG signals.

### 3.7. Data Segmentation Algorithm

**Figure 8:**
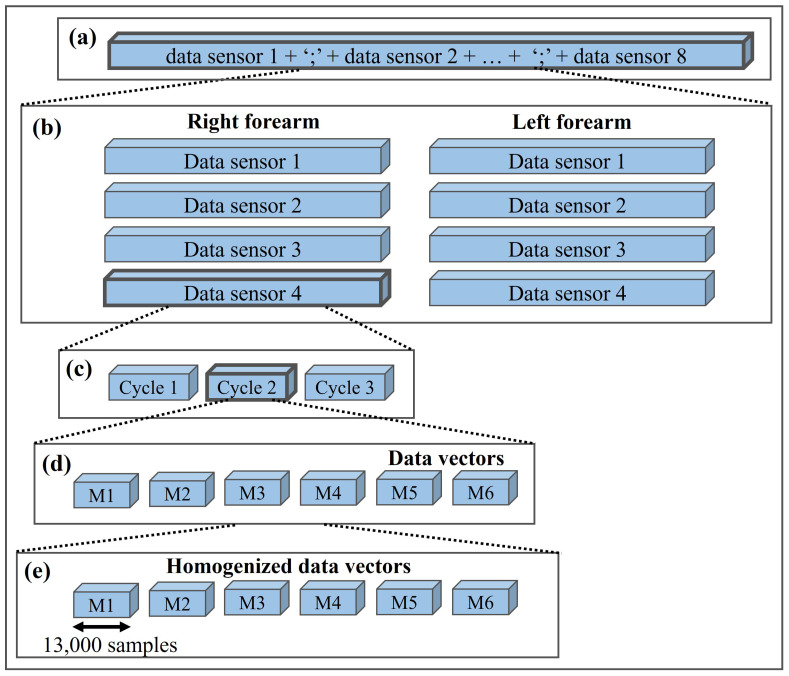
Schematic representation of the steps considered in the division algorithm. (**a**) $DS_{j}$ complete vector, (**b**) Division of the $DS_{j}$ vector into each of the eight sensors, (**c**) Division of each data sensor into each of the three cycles, (**d**) Division of each cycle into each of the six movements, (**e**) Homogenization of the each movement data.

To obtain homogeneous vectors of each of the movements from the sEMG signals registered through the GUI, a division algorithm was implemented in the Python environment. Figure 8 shows a general scheme with the 5 stages considered by the division algorithm. The first step consisted of loading the vector that has the information of the 8 sensors separated by semicolons. The structure of the mentioned vector can be observed in Figure 8a). As second step, the vector was divided into 8 subvectors (4 for the left forearm and 4 the right), this step is represented in Figure 8b).

The next step consists of obtaining each of the 3 cycles per sensor, represented in Figure 8c). To this end, the division algorithm used the data recorded by the sensor located in the upper part of the forearm (sensor 1). This sensor was selected since it measured higher amplitudes in the extension movement than the other movements, establishing a reference for the division. Through the location of these maximums, the algorithm compute the data between the last peak and the end of the vector, thus determining the average number of data per cycle without counting data obtained at the intermediate times when a new reply started. Step 4 of the division algorithm consists of obtaining the 6 movements per cycle, that is one vector for each motion. This part of the process is represented in Figure 8d). Then, a homogenization process was executed to obtain 6 vectors of 13,000 data points. In this process, the algorithm calculates the difference between the length of the motion vector and 13,000 samples. Then, the half of the difference in the beginning of the vector is cut, as is half in the end. Finally, the information of each motion was stored in CVS files with specific labels according to the movement to which they corresponded. The last step of the division algorithm is represented in Figure 8e).

### 3.8. EMG Signals Classification

For the validation of the WyoFlex bands operation a classification algorithm based on ANNs in Matlab was implemented through the Neural Net Pattern Recognition toolbox. This toolbox has a two-layer feedforward network with hidden sigmoid neurons and softmax output neurons. Note that the data set for the classification algorithm considers 15 participants who executed 3 cycles of 6 movements with each of their forearms. Therefore, the data set is made up of 540 input signals to the ANNs (90 signals per movement).

In order to evaluate the classification of the hand movements with the minimum processing of the signals, complete vectors corresponding to the calculation of the Fast Fourier Transform (FFT) of each sensor and vectors generated from the calculations of the average of the FFT in data segments contained in 10 Hz, were used as input in the ANN. In the first case, the Matlab function fft() was used to calculate the FFT, taking the first half of the obtained vector as input. Subsequently, Algorithm 1 described below was implemented in the other case.
**Algorithm 1** Average of sequences of the signal FFT**Input:**dataSensor**Output:**vectorAveragesFFT1:**Start**2:**Initialization**3:vectorAveragesFFT←zeros(1,50);4:startValue←5;5:endValue←130;6:i←50;7:vectorFFT←abs(fft(dataSensor));8:**for**(i=1→50)**do**9:      averageFFT←mean(vectorFFT(startValue:endValue));10:    startValue←startValue+130;11:    endValue←endValue+130;12:    vectorAverageFFT(1,i)←averageFFT;**   Return**vectorA verageFFT

Note that the first five samples of the signal were not considered to obtain the FFT vector and the vector with the averages of the FFT segments due to the first five samples corresponding to the DC value frequency components, which is not considered valuable information for the movement classification process. Once the FFT and the average of the sequences of the FFT have been obtained (for the 4 sensors of every movement), the input matrix for the ANNs is defined. For the first case, the input matrix satisfies the following structure

(2) $$In_{a}={\left[aFFT_{1},aFFT_{2},aFFT_{3},\cdots ,aFFT_{540}\right]}^{⊤},$$

where each component of $In_{a}$ corresponds to a vector of the concatenation of the FFT of the 4 sensors for each motion. Then, the dimensions of the input matrix are 540× 26,000.

On the other hand, in the second case the input matrix for the ANNs is given by

(3) $$In_{b}={\left[vectorAverageFFT_{1},vectorAverageFFT_{2},\cdots ,vectorAverageFFT_{540}\right]}^{⊤},$$

each component of $In_{b}$ corresponds to a vector of the concatenation of vectorAverageFFT of each sensor. Then, the dimensions of this matrix are 540×200. For both cases (vectors defined in (2) and (3)), were evaluated with three different numbers of neurons (34, 49, and 63). The obtained results are presented in the subsequent section.

## 4. Results

**Figure 9:**
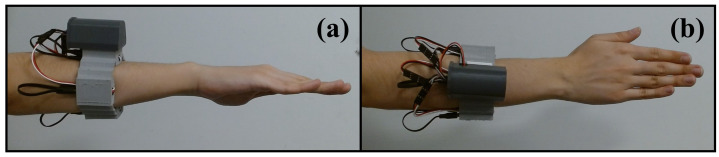
WyoFlex band. (**a**) Lateral view of the location of the Wyoflex band in the forearm, (**b**) Superior view of the location of the Wyoflex band in the forearm.

This section evaluates the proposed WES integrated by 2 WyoFlex bands. Figure 9a,b show the lateral and superior views of the right forearm, respectively. In this case, the WyoFlex band is located in the middle of the forearm. Notice that the manufactured WyoFlex band satisfies all the design criteria mentioned in Section 2.

**Figure 10:**
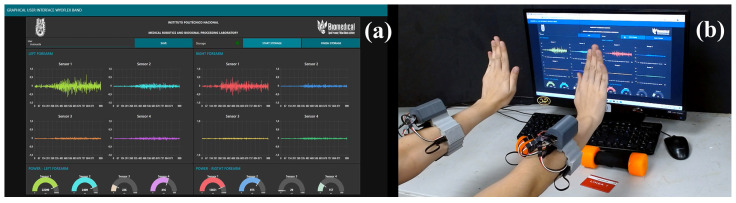
Example of the extension movement in the test protocol. (**a**) Visualization of the sEMG signals in the GUI, (**b**) Example of the extension movement.

Once the WyoFlex bands were located in the forearms of each participant, the test protocol described in Section 3.3 was executed to acquire the sEMG signals. Figure 10 shows an example of the execution of the extension movement. In this case, the proposed GUI is evidenced in Figure 10a), where the 8 sEMG signals are displayed aside from their corresponding power in mJ. Additionally, the green LED shows the correct function of the storage process. On the other hand, in Figure 10b), the forearm of the participant is shown executing the extension movement. The execution of the complete test protocol can be consulted as supplementary material in the following link: https://www.dropbox.com/s/1qp3d0y0fmtyb1h/GUI%20VIDEO%204K.mp4?dl=0 (accessed on 30 July 2022).

**Figure 11:**
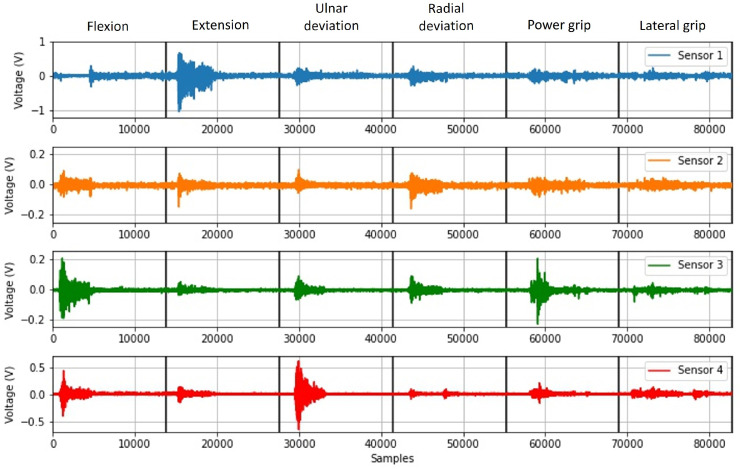
Signals obtained from each of the four sEMG sensors with the signal acquisition protocol.

Subsequently, the signals observed in Figure 11 were obtained from the signal acquisition protocol for each repetition. This figure shows the signals acquired for the four sensors. Then, these signals were composed of the six movements, which, based on the implementation of the division algorithm, were segmented into subvectors determined by the solid black vertical lines.

Figure 11 shows that the sEMG signal with the maximum amplitude is obtained from Sensor 1. This signal varies from −1 V to 0.8 V in the section corresponding to the execution of the extension movement. In the case of Sensor 2, the maximum amplitude is obtained in the radial deviation movement, where the signal varies from −0.18 V to 0.12 V. On the other hand, in sensors 3 and 4, the maximum amplitudes vary in the ranges of −0.20 V to 0.22 V and −0.53 V to 0.53 V, corresponding to the movements of power grip and ulnar deviation, respectively.

**Figure 12:**
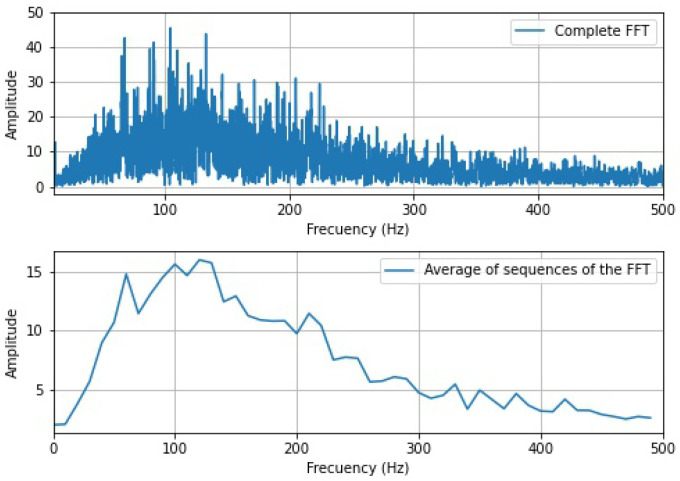
FFT signals used in the ANNs.

Then, as part of the implementation of the classification algorithm, the computation of the FFT and the vector of averages of the FFT for each sensor was carried out, which is evidenced in Figure 12. The signal which contains the complete spectrum of the Fourier transform was conformed of 6500 samples, subsequently forming input vectors of 26,000 samples. On the other hand, the vector corresponding to the FFT average of the sequences has only 50 samples, considering that a representative value was calculated for ranges of 10 Hz. This generated input vectors of 200 samples for the ANNs.

**Figure 13:**
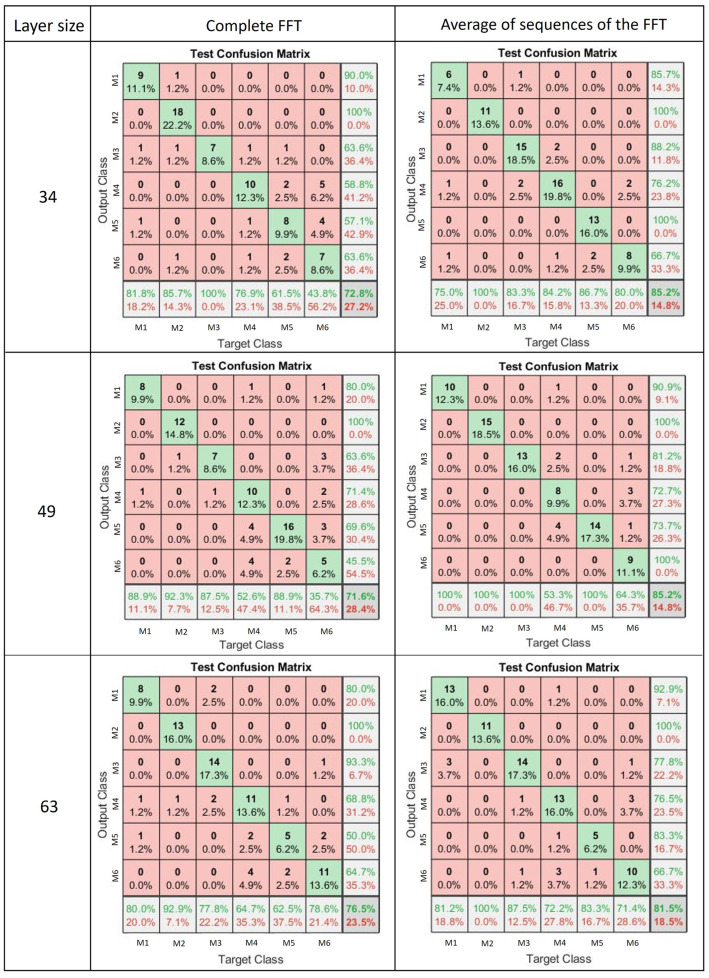
Results test for the ANN.

Once the Neural Net Pattern Recognition toolbox of Matlab was implemented to evaluate two vector inputs (vector $In_{a}$ and $In_{b}$), the confusion matrices given in Figure 13 were obtained for each of the three configurations of the ANNs. That is, both vectors ($In_{a}$ and $In_{b}$) were evaluated with 34, 49, and 63 neurons in the hidden layer. Figure 13, in its first column, shows the number of neurons considered in the hidden layer. The second column shows the obtained confusion matrices for each of three cases (34, 49, and 63 neurons in the hidden layer) when the vector $In_{a}$ serves as input information of the ANN. In contrast, the third column evidenced the confusion matrices for each case (34, 49, and 63 neurons in the hidden layer) when the vector $In_{b}$ serves as input of the ANNs.

Here, the test confusion matrices obtained with the complete FFT show an overall recognition accuracy of 72.8%, 71.6%, and 76.5% for the layer size of 34, 49, and 63, respectively. Notice that with the layer size equal to 49, the gesture M2 (“Extension”) is the one with the highest sensitivity (92.3%), whereas the gesture M6, which corresponds to the “Lateral grip” is the one with the lowest (35.7%). Regarding precision, the gesture M2 (“Extension”) has a perfect result (100%).

On the other hand, the test confusion matrices obtained with the FFT average sequences show an overall recognition accuracy of 85.2%, 85.2%, and 81.5% for the layer sizes of 34, 49, and 63, respectively. In the case of the layer size being equal to 49, the gestures M1 (“Flexion”), M2 (“Extension”), M3 (“Ulnar deviation”), and M5 (“Power grip”) are the ones with the highest sensitivity (100%). In contrast, the gestures M4 (“Radial deviation”) and M6 (“Lateral grip”) are the two with the lowest. Regarding precision, the gestures M2 (“Extension”) and M6 (“Lateral grip”) have a perfect result (100%).

In addition, the values for the training and validation stages were obtained, as can be observed in Table 3 and Table 4. Moreover, the general performance of the ANNs is reported. Additionally, the number of epochs executed for each case was indicated, as well as the time that the complete process of the ANNs took in the Matlab toolbox.

Notice that the values with the complete FFT generated better results for the training stage. At the same time, the other two cases (validation and test) had a better performance in the case of signals obtained from the average of segments of the FFT. In addition, the computational time is considerably higher in the case of the complete FFT, since each of the input signals has more samples, generating a higher computational cost.

## 5. Conclusions

This paper presents the development of a WES integrated by 2 WyoFlex bands. The proposed design of the WyoFlex band is manufactured using 3D printing techniques. Moreover, using materials such as TPU and PLA guarantees the creation of a wearable device, which permits the acquisition of 4 sEMG signals from the forearm. Then, a dataset integrated by 540 sEMG signals acquired from 15 volunteers was obtained. The dataset was validated by implementing a classification algorithm based on the Neural Net Pattern Recognition toolbox of Matlab. The proposed ANNs configuration evidenced a classification accuracy of 85% when the FFT average sequences were used.

## 6. Patents

A derivative from this work is currently undergoing software registration process.

## Figures and Tables

**Table 1:** Characteristics of sEMG Armbands.

	Myo Armband	Two-Channel Armband	3DC Armband	gForce	WyoFlex
	**(Thalmic Lab)**	**(M. Tavakoli)**	**(Laval University)**	**(OYMotion)**	
sEMG channels	8	2	10	8	4
ADC	8 bits	-	10 bits	8 bits	12 bits
Max sampling rate	200 Hz	1000 Hz	1000 Hz	1000 Hz	1600 Hz
Thickness	1.15 cm	-	1.6 cm	1 cm	1.55 cm
Weight	93 g	-	62 g	80 g	150 g
Price	475 USD	-	150 USD	1250 USD	250 USD

**Table 2:** Printing times.

Piece	Time
Flexible band	29 h 58 min
Sensor case (2)	1 h 15 min
Sensor case (3)	43 min
Microcontroller case	4 h 57 min
Microcontroller cover case	18 min

**Table 3:** Results of the ANNs with the complete FFT.

Laye Size	34	49	63
Training	94.2%	100.0%	94.2%
Validation	64.2%	61.7%	71.6%
Test	72.8%	71.6%	76.5%
Overall performance	86.5%	90.0%	88.1%
Time (s)	19	31	33
Epochs	91	115	103

**Table 4:** Results of the ANNs with the averages of sequences of the FFT.

Laye Size	34	49	63
Training	81.5%	91%	93.1%
Validation	76.5%	80.2%	88.9%
Test	85.2%	85.2%	81.5%
Overall performance	81.3%	88.5%	90.7%
Time (s)	1	2	2
Epochs	91	109	134

## Data Availability

Not applicable.

## Supplementary Materials

The following are available online at https://www.mdpi.com/article/10.3390/s22165931/s1, Video S1: sensors-22-05931-supplementary.

## Abbreviations

The following abbreviations are used in this manuscript:

sEMG	Surface Electromyography
IoT	Internet of Things
WES	Wearable Electromyographic System
3D	Three-Dimensional
EMG	Electromyography
ANNs	Artificial Neural Networks
GUI	Graphical User Interface
UDP	User Datagram Protocol
CAD	Computer-Aided Design
TPU	Thermoplastic Polyurethane
PLA	Polylactic Acid
Li-Po	Lithium-Polymer
ADC	Analog-Digital Converter
